# Supplementary material for: Transparent and high-porosity aluminum alkoxide network-forming glasses
Source: Nat Commun. 2024 Aug 26;15:7339. doi: 10.1038/s41467-024-51845-1 (PMC11347621; doi:10.1038/s41467-024-51845-1)
Supplement: Supplementary file 1 — Supplementary Information [file 41467_2024_51845_MOESM1_ESM.pdf]

Supplementary Information

Transparent and High-porosity Aluminum Alkoxide Network-forming Glasses

Zihui Zhang[1], Yingbo Zhao[1,2,*]

[1]*School of Physical Science and Technology, ShanghaiTech University, Shanghai, 201210, P. R. China.*
[2]*Shanghai Key Laboratory of High-Resolution Electron Microscopy, ShanghaiTech University, Shanghai 201210, China.*
*Corresponding authors: zhaoyb2@shanghaitech.edu.cn

Supplementary Note 1

Materials

Bis(2-hydroxyethyl) terephthalate (BHET) were bought from Sinopharm Wokai. (Methanetetrayltetrakis(benzene-4,1-diyl))tetramethanol (MTBT), 1,1,1-triphenyl-2,5,8,11-tetraoxatridecan-13-ol (TPTO) were bought from Yanshen Technology. Ethanol absolute (EtOH), 1-butanol (*n*-BuOH), glacial acetic acid (HOAc), tetrahydrofuran (THF), ethylene glycol methyl ether (EGME), acetone (AC), methanol anhydrous (MeOH) were bought from Sinopharm Shanghai Trial. Aluminum sec-butoxide (Al-(O*s*Bu)$_3$) were bought from Bidepharm. All reagents purchased were used directly without further purification.

Instruments and methods

Powder X-ray diffraction is measured with Rigaku MiniFlex600-C X-ray diffractometer with Cu target. Mettler Toledo TGA/DSC 3+ was used for TGA testing under a nitrogen atmosphere with a flow rate of 50.0 mL/min, a heating rate of 20 K/min, and a measurement range of 30-800 °C. Rheological measurement was conducted using an Anton Paar Modular Compact Rheometer MCR 302, with a flat rotor PP25 measuring system measured at the temperature of 25 °C. The shear strain was increased logarithmically at a fixed angular frequency of 10 rad/s.

Elemental analysis for oxygen content was carried out on a Perkinelmer SERIES ll 2400 (Clarus 580) elemental analyzer, and the routine C,H,N measurement was carried out with Elementar Vario EL CUBE fully automatic elemental analyzer. The Al element content was determined using Thermo ICP-OES Icap7400 Plasma Emission Spectroscopy. The scanning electron microscopy images and energy dispersive X-ray spectroscopy of the samples were captured using JSM-7800F Prime scanning electron microscope with an Oxford EDS accessory.

The Agilent Cary 5000 UV visible near-infrared spectrophotometer was used to measure diffuse reflectance and absorption; attenuated total reflection-Fourier transform infrared spectroscopy (ATR-FTIR) were collected on PerkinElmer Frontier in the range of 4000-400 cm^{-1} at a resolution of 2 cm^{-1}. The fluorescence of Al-MTBT glass was acquired using HORIBA Fluorolog-3 photoluminescence spectrometer with 320 nm excitation.

N_2 uptake at 77 K was measured using a Quantachrome Instruments Autosorb-iQ-MP-AG BET gas adsorption specific surface analyzer. The instrument software VersaWin was used to calculate the BET specific surface area and DFT pore size distribution for all adsorption isotherms. The Anton Paar Ultrapyc 5000 was used to determine the sample's skeleton density. NanoTest Vantage (MML, UK) was used for nanoindentation hardness testing with a Berkovich indenter BBF-68.

Synthesis of UiO-66@Al-BHET

UiO-66 is synthesized according to literature reports [supplementary ref. 1]. The UiO-66 is added to the above-mentioned Al-BHET synthesis (200 μL Al-BHET solution per 8 mg UiO-66) and the evaporation vitrification is carried out same as Al-BHET.

Supplementary Table 1. Name, quantity, and feeding percentage of the sample

Number	Name	Feed ratio of Al to hydroxyl groups
No 1.	Al-BHET	1:2
No 2.	Al-BHET-TPTO	1:2 (n_{OH}BHET:n_{OH}TPTO= 9:1)
No 3.	Al-BHET-TPTO-2	1:2 (n_{OH}BHET:n_{OH}TPTO= 2:1)

Supplementary Table 2. Skeleton density test

Sample Name	Sample weight	Skeleton density	Average volume	Relative average. deviation.
Al-BHET	0.1232 g	1.4108 g/cm^3	0.0873 cm^3	0.1878 %
Al-BHET-TPTO	0.1204 g	1.4646 g/cm^3	0.0822 cm^3	0.2636 %
Al-MTBT	0.1066 g	1.3337 g/cm^3	0.0799 cm^3	0.2205 %

Supplementary Table 3. Pore volume calculated from gas adsorption analysis

Sample Name	Surface Area (77K N_2)	Pore volume (195 K CO_2)	Porosity (195 K CO_2)
Al-BHET	/	0.1067 cc/g	13.08 cm^3/cm^3
Al-BHET-TPTO	496.904 m^2/g	0.2949 cc/g	30.16 cm^3/cm^3
Al-MTBT	362.544 m^2/g	0.2200 cc/g	22.69 cm^3/cm^3

Supplementary Table 4. Summary of elemental analysis

Sample Name	C/%	H/%	O/%	Al/%
Al-BHET	43.1	4.9	42.8	10.3
Al-BHET-TPTO	36.7	4.7	43.7	11.9
Al-MTBT	43.2	5.6	39.8	12.4

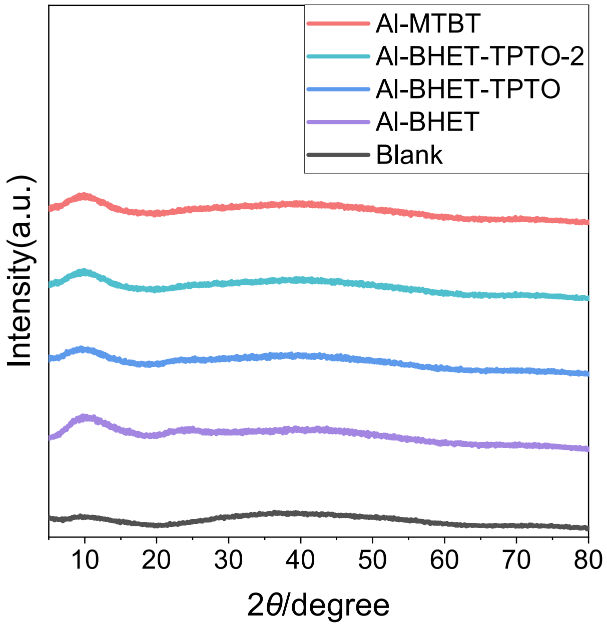

Supplementary Figure 1. X-ray diffraction pattern of aluminum alkoxide glasses and monoliths. The absence of diffraction peaks for Al-BHET, Al-MTBT, Al-BHET-TPTO and Al-BHET-TPTO-2 confirms their amorphous nature.

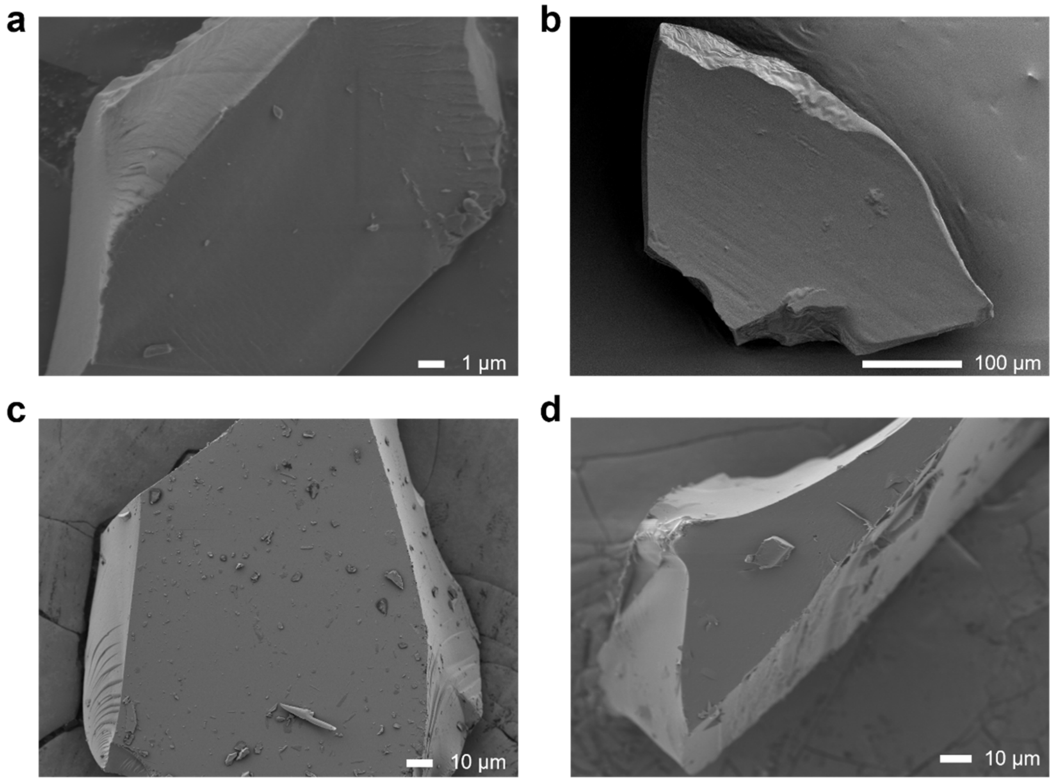

Supplementary Figure 2. Scanning electron microscopy image of aluminum alkoxide glasses and monoliths. **a**, Al-BHET; **b**, Al-MTBT; **c**, Al-BHET-TPTO; **d**, Al-BHET-TPTO-2.

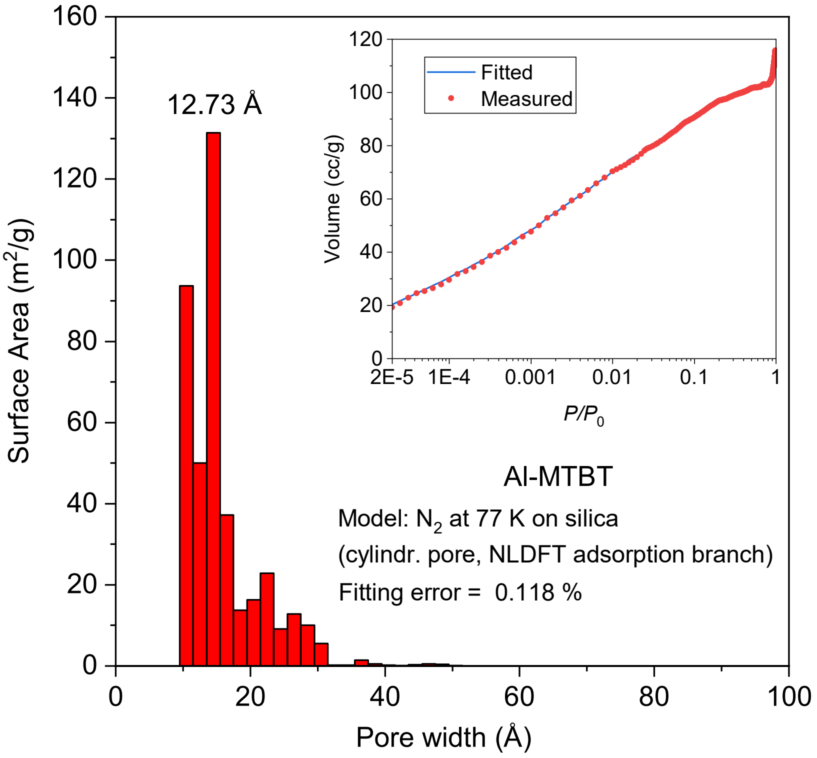

Supplementary Figure 3. Pore size distribution of Al-MTBT under 77 K nitrogen gas. Fitting of the N_2 adsorption isotherm of Al-MTBT showing its microporosity.

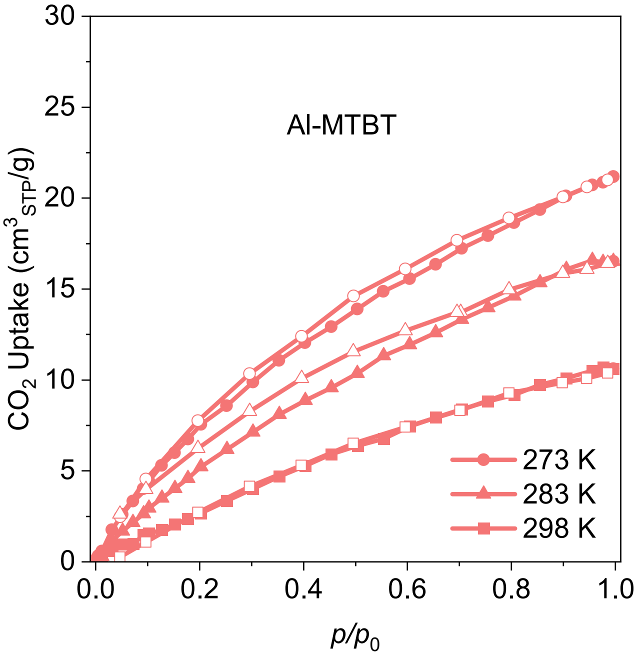

Supplementary Figure 4. CO_2 adsorption for Al-MTBT. The CO_2 uptake at different temperatures also confirms the porosity of the Al-MTBT.

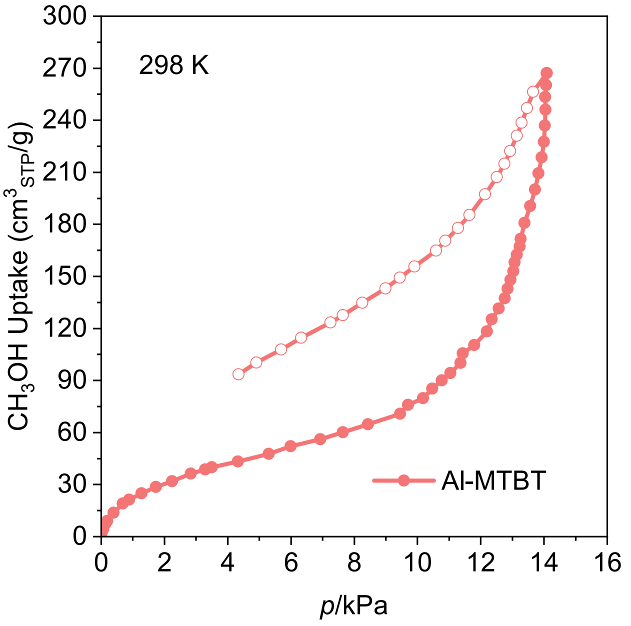

Supplementary Figure 5. 298 K methanol vapor adsorption of Al-MTBT. The methanol uptake showing the porosity of the Al-MTBT.

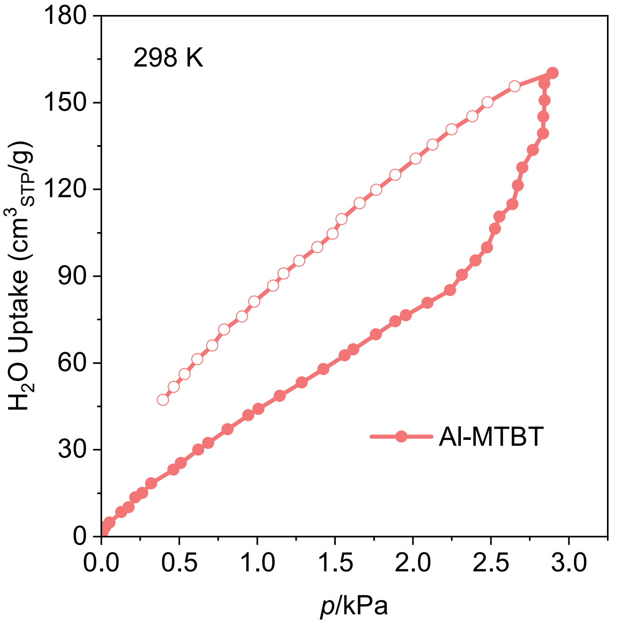

Supplementary Figure 6. 298 K water vapor adsorption of Al-MTBT. The water uptake showing the porosity of the Al-MTBT.

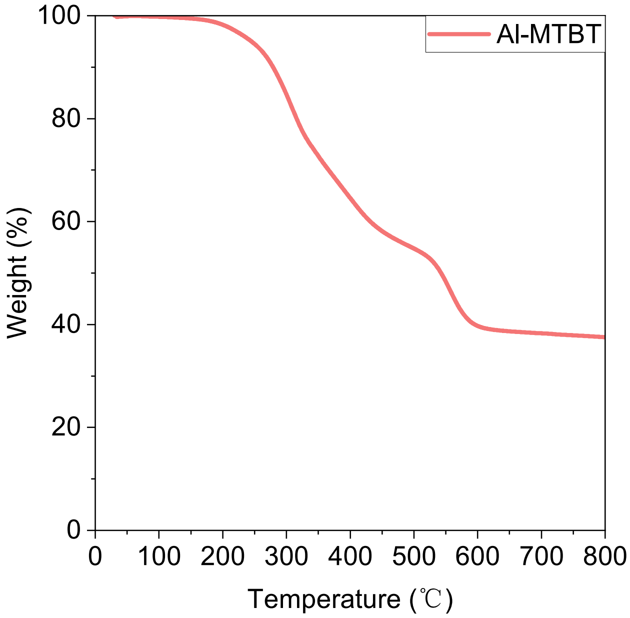

Supplementary Figure 7. TGA curve of Al-MTBT. The Al-MTBT shows thermal stability up to 150 °C without weight loss.

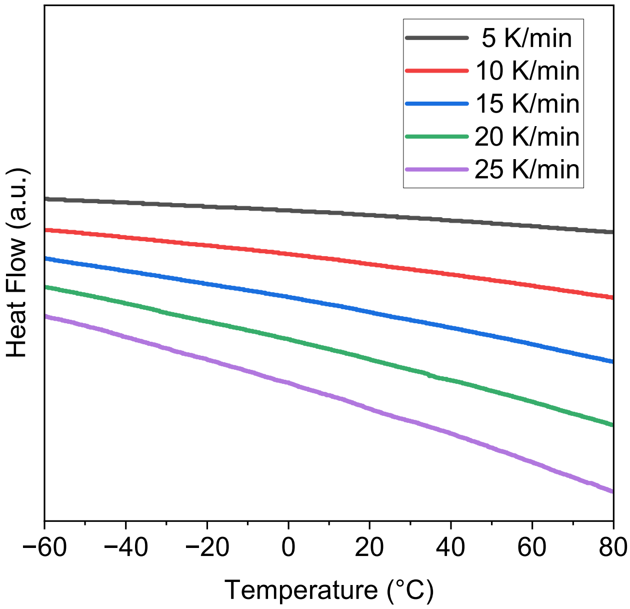

Supplementary Figure 8. DSC curve of as-synthesized Al-MTBT. No glass transition is observed for scan rates varied from 5 K/min to 25 K/min.

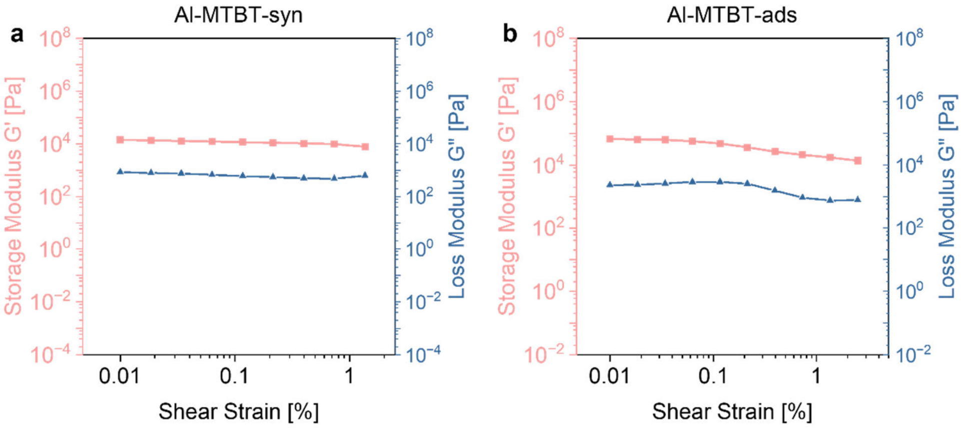

Supplementary Figure 9. Rheology measurement for Al-MTBT. a As-synthesized Al-MTBT. **b** Activated Al-MTBT. The suffix "syn" denote the as-synthesized sample, and "ads" denote the activated sample.

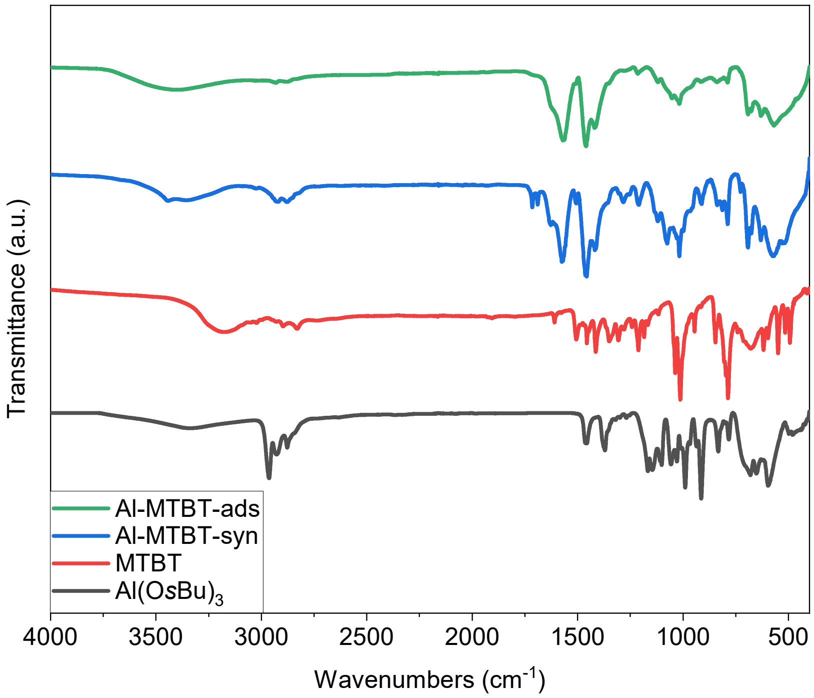

Supplementary Figure 10. Infrared spectrum of Al-MTBT. The diminished absorption above 3000 cm^{-1} shows deprotonation of the MTBT linker.

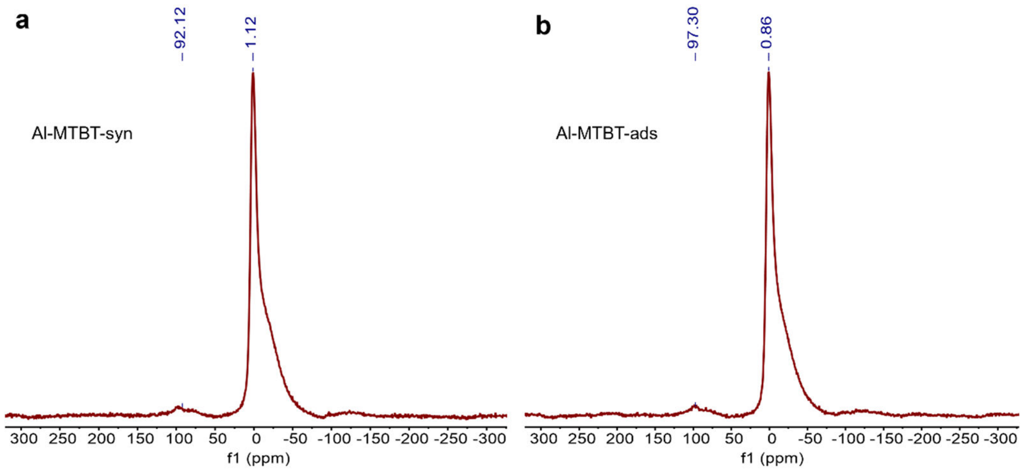

Supplementary Figure 11. ^{27}A1 CPMAS NMR spectra of Al-MTBT. a As-synthesized sample. **b** Activated sample. The suffix "syn" denote the as-synthesized sample, and "ads" denote the activated sample.

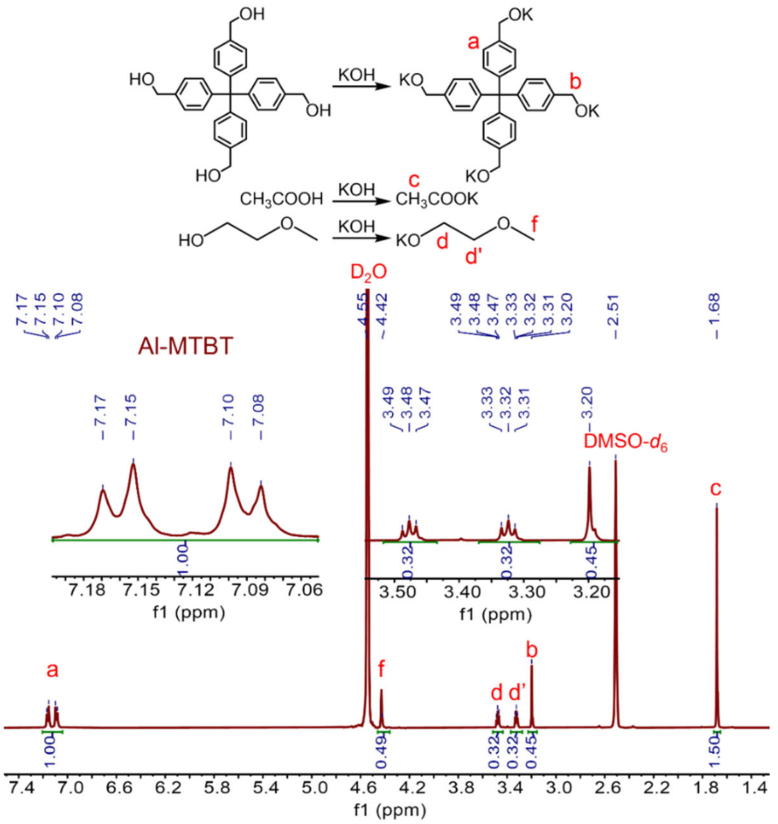

Supplementary Figure 12. Digested NMR spectrum of Al-MTBT (using a mixed solvent of DMSO-*d*$_6$ and D_2O, with the addition of KOH). Al-MTBT, ^{1}H NMR (500 MHz, DMSO-*d*$_6$) δ 7.21-7.04 (m, 12H), 4.42 (s, 3H), 3.48 (t, *J* = 5.3 Hz, 2H), 3.32 (t, *J* = 5.2 Hz, 2H), 3.20 (s, 8H), 1.68 (s, 3H). From the integration of H, The molar ratio MTBT: HOAc: EGME=0.12: 1: 0.32.

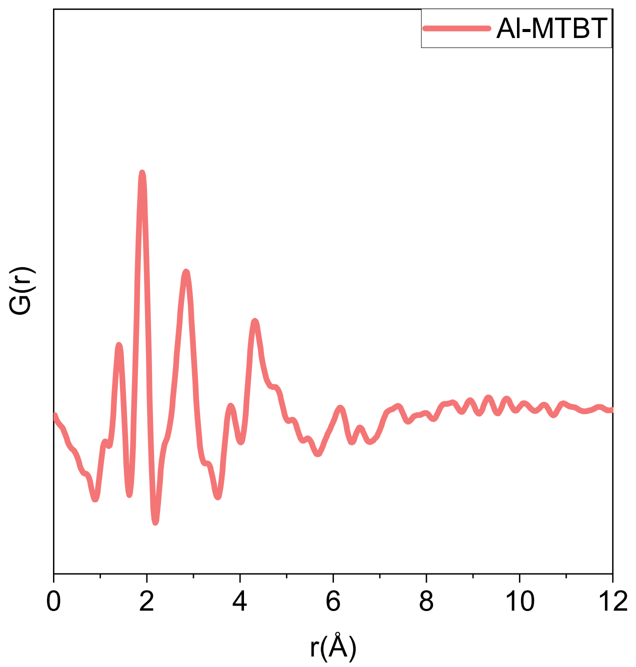

Supplementary Figure 13. Pair-distribution function of Al-MTBT. The short-range ordering is consistent with the structure of AlOCs.

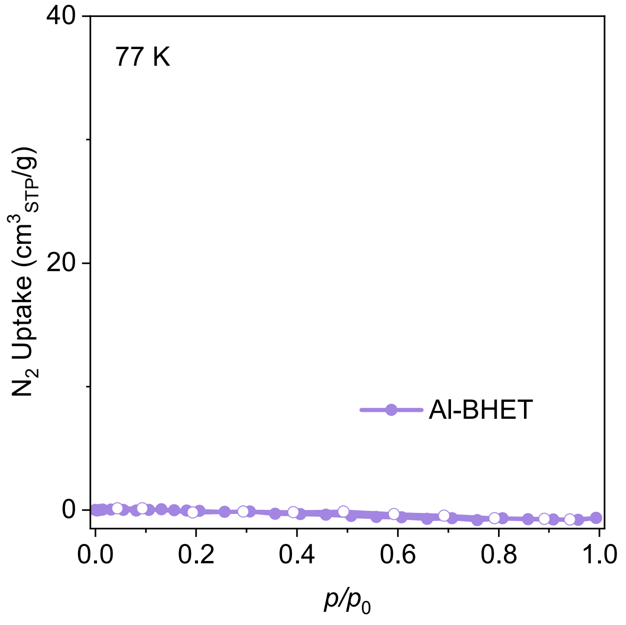

Supplementary Figure 14. 77 K N_2 adsorption of Al-BHET. The small pores of Al-BHET give negligible nitrogen uptake.

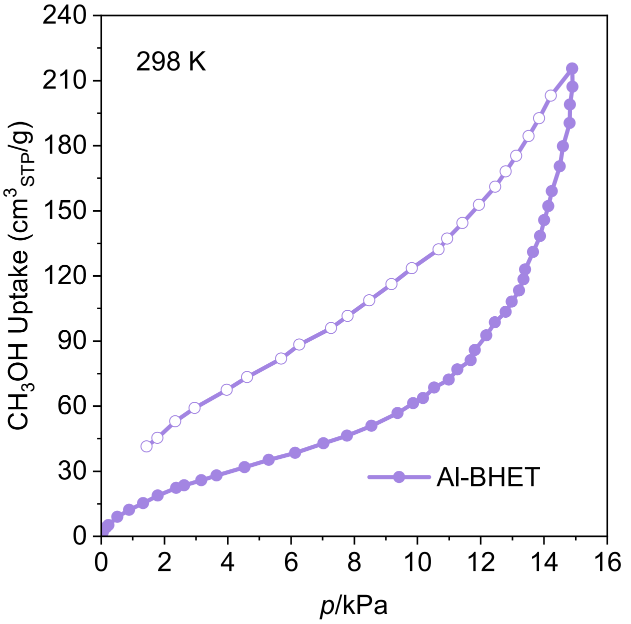

Supplementary Figure 15. 298 K methanol vapour adsorption of Al-BHET. The methanol uptake showing the porosity of the Al-BHET.

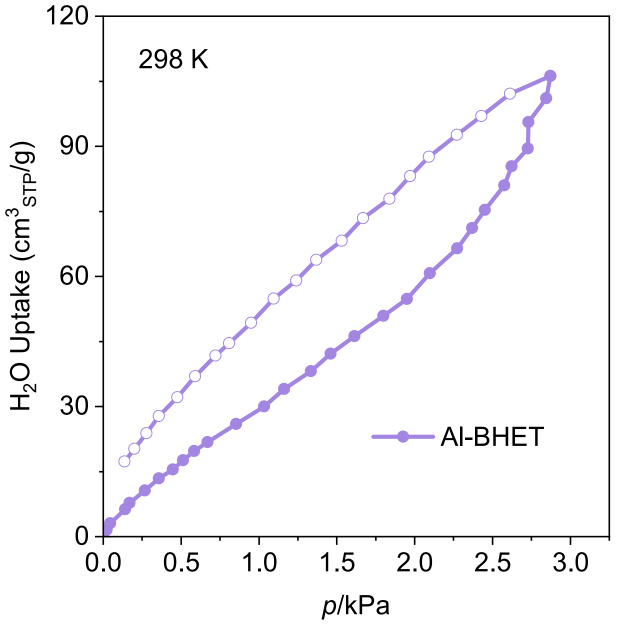

Supplementary Figure 16. 298 K water vapor adsorption of Al-BHET. The water uptake showing the porosity of the Al-BHET.

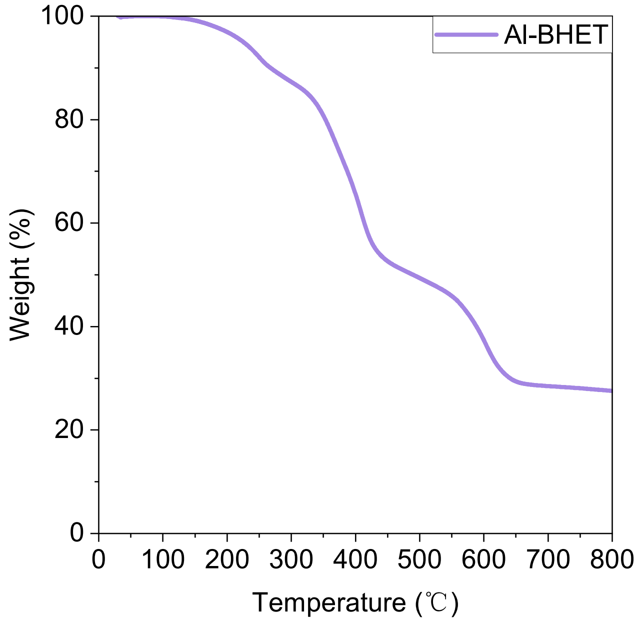

Supplementary Figure 17. TGA curve of Al-BHET. The Al-BHET shows thermal stability up to 150 °C without weight loss.

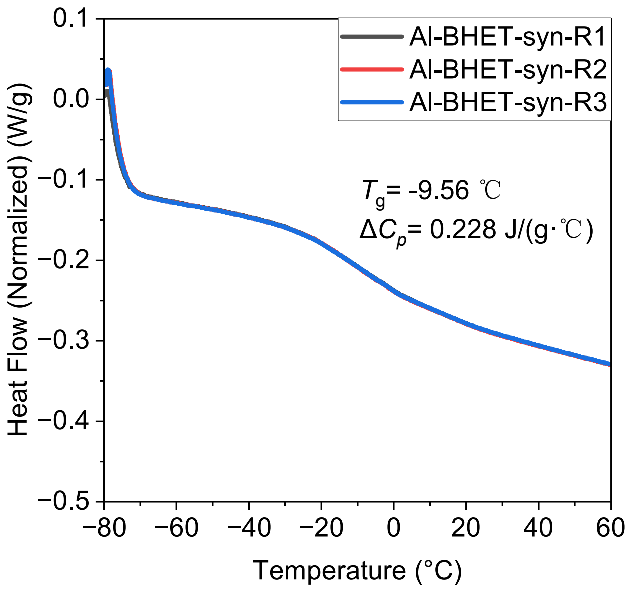

Supplementary Figure 18. DSC repeatability test for as-synthesized Al-BHET. The Up and down scan rates are both 20 K/min, the suffix "syn" denote the as-synthesized sample, R1 to R3 denote the number of cycles.

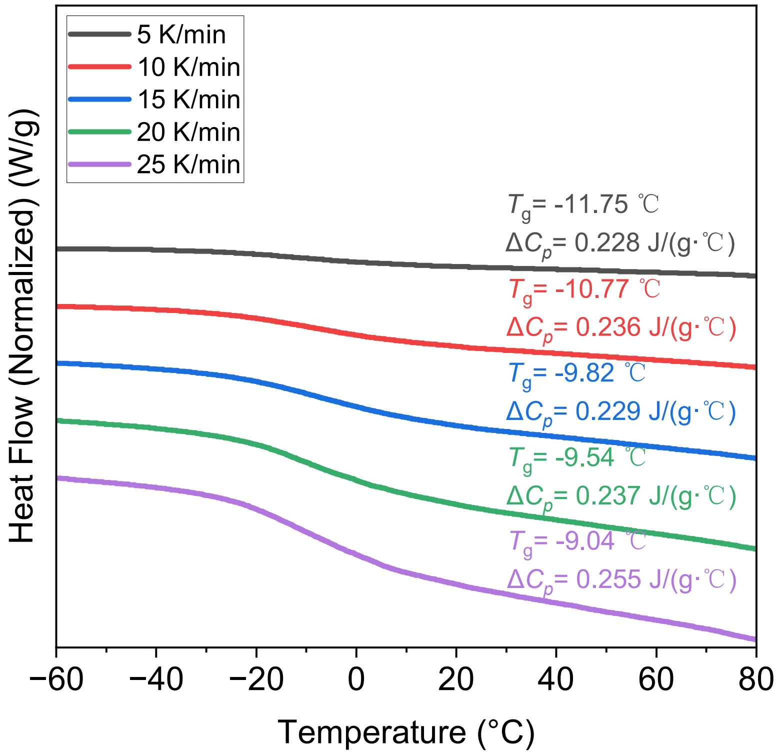

Supplementary Figure 19. DSC with different up scan rate for as-synthesized Al-BHET. The increase of fictive temperature with higher scan rate confirms the presence of glass transition.

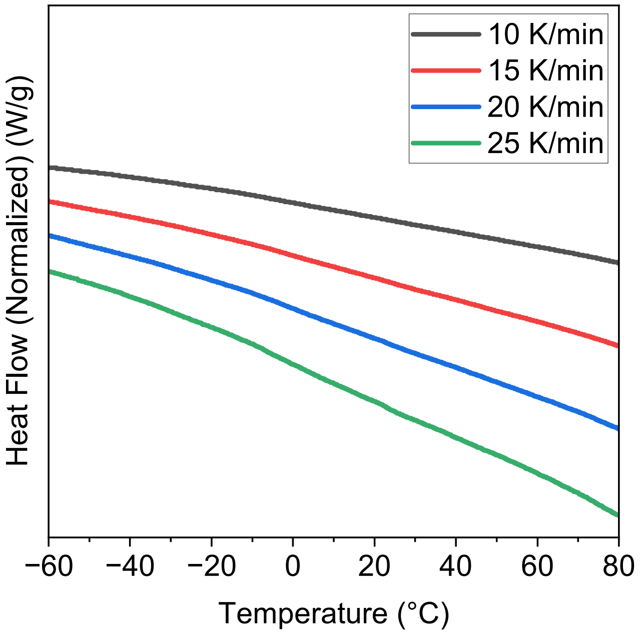

Supplementary Figure 20. DSC with different up scan rate for activated Al-BHET. No glass transition is observed for scan rates varied from 10 K/min to 25 K/min.

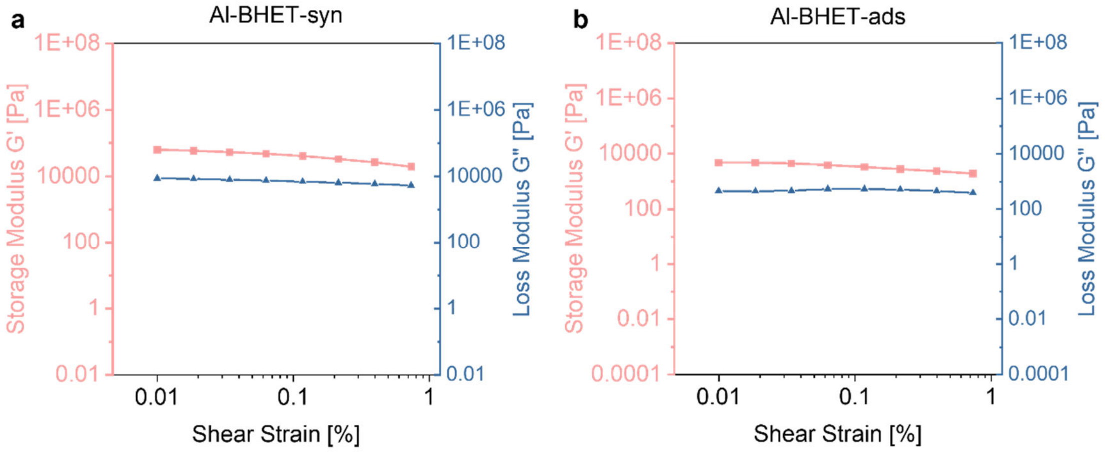

Supplementary Figure 21. Rheology measurement for Al-BHET. a As-synthesized Al-BHET. **b** Activated Al-BHET. The suffix "syn" denote the as-synthesized sample, and "ads" denote the activated sample.

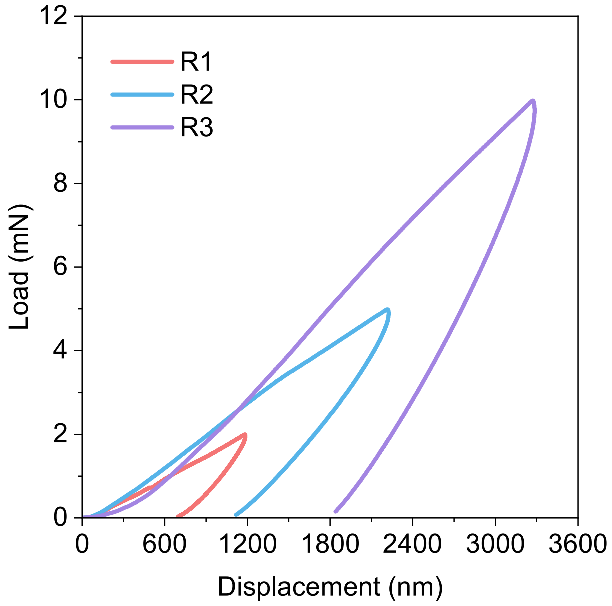

Supplementary Figure 22. Nano indentation test of Al-BHET. The measurement shows the Al-BHET is an elastic solid.

Supplementary Table 5. Nano indentation test results for Al-BHET

**Runs**	**H_{IT} /MPa**	**E_{IT} /GPa**	**E^* /GPa**
**1**	80.895	1.0893	1.197
**2**	64.472	0.64147	0.70491
**3**	55.058	0.66617	0.73026

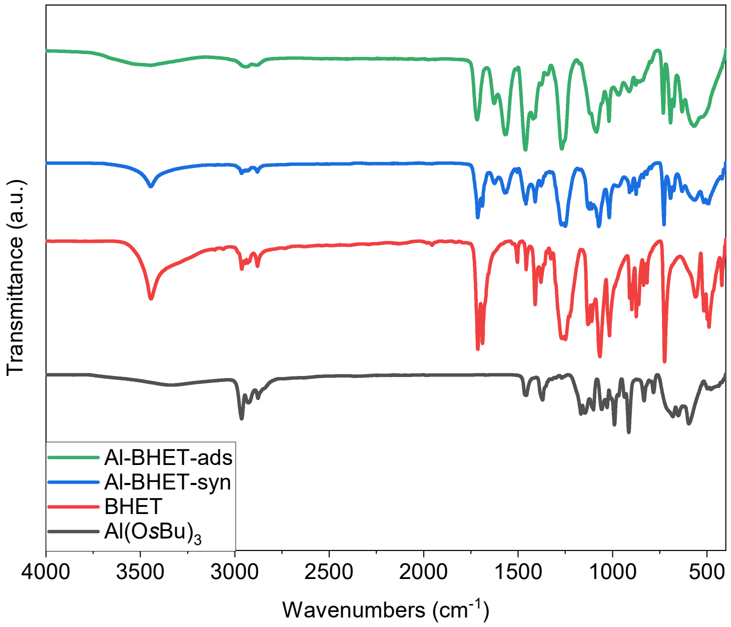

Supplementary Figure 23. Infrared spectrum of Al-BHET. The diminished absorption above 3000 cm^{-1} shows deprotonation of the BHET linker.

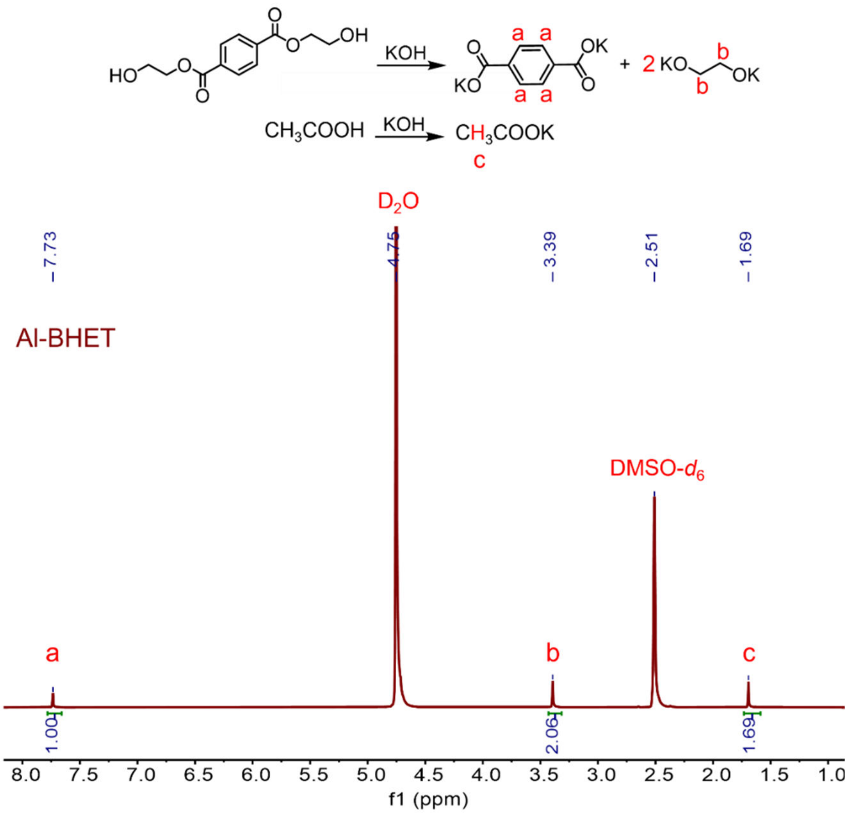

Supplementary Figure 24. Digested NMR spectrum of Al-BHET. ^{1}H NMR (500 MHz, DMSO-d_6) δ 7.73 (s, 4H), 3.39 (s, 8H), 1.69 (s, 3H). According to the integration of peak area, the molar ratio HOAc: BHET= 2.25.

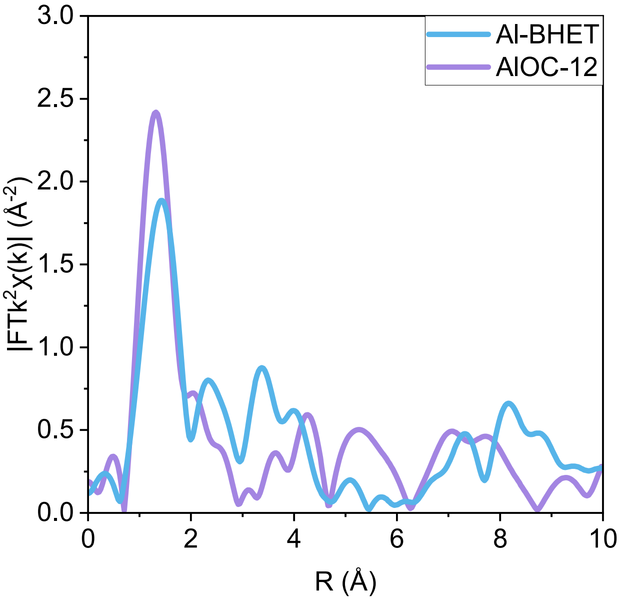

Supplementary Figure 25. R-space plot from the EXAFS of Al-BHET. The consistency of the first-shell for Al-BHET and AlOC-12 supports their structural similarity

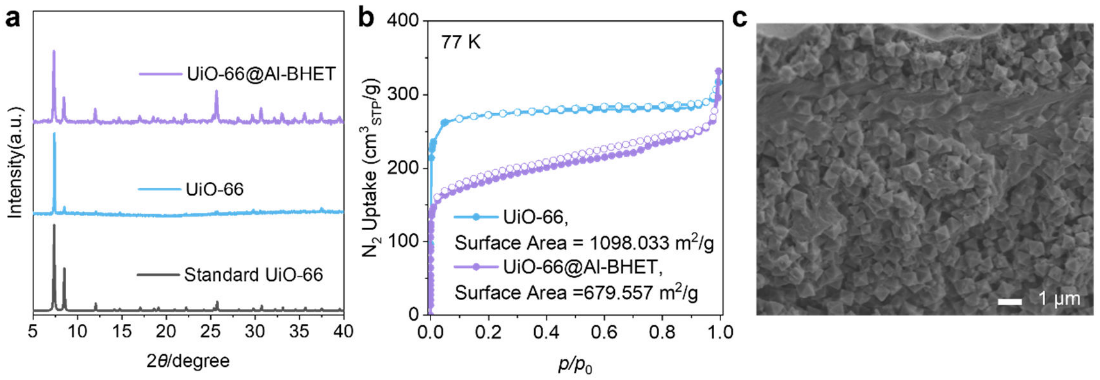

Supplementary Figure 26. Characterization of UiO-66@Al-BHET composite materials. **a** XRD patterns of UiO-66 and UiO-66@Al-BHET. **b** N_2 adsorption isotherms of UiO-66 and UiO-66@Al-BHET composite materials at 77 K. **c** SEM images of UiO-66@Al-BHET.

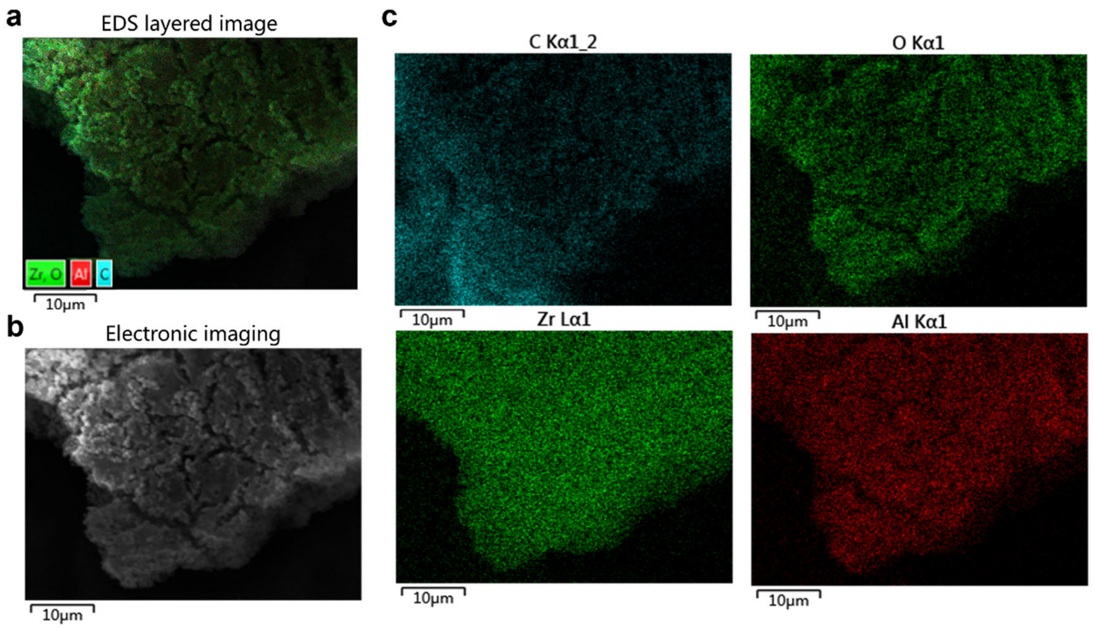

Supplementary Figure 27. Scanning electron microscope energy-dispersive X-ray spectroscopic mapping of UiO-66@Al-BHET with an accelerated voltage of 20 kV. **a** Energy-dispersive X-ray spectroscopic mapping layered image. **b** scanning electron microscopy image. **c** Energy-dispersive X-ray spectroscopic mapping of various element showing their spatial distributions.

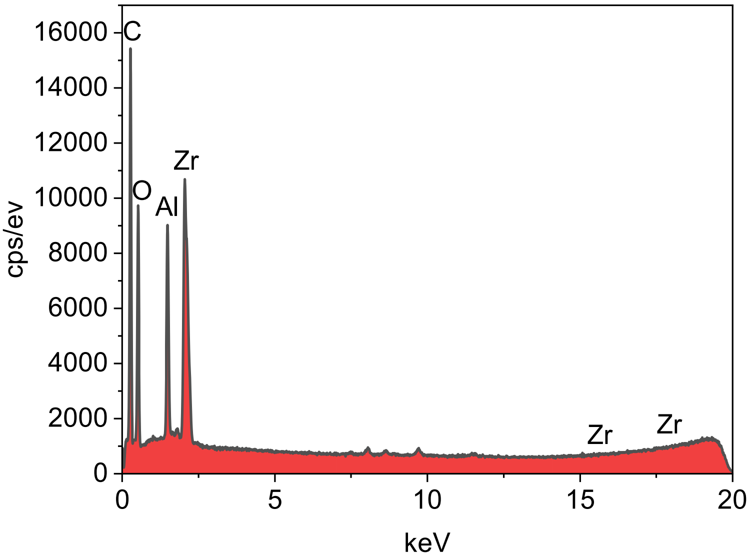

Supplementary Figure 28. UiO-66@Al-BHET EDS with an accelerated voltage of 20 kV. The prescence of both Zr and Al element is confirmed, which are used for mapping the distribution of UiO-66 in Al-BHET.

Supplementary Table 6. EDS results of UiO-66@Al-BHET

Spectral labels	Weight percentage
C	51.53
O	29.59
Al	5.03
Zr	13.85
Total	100.00

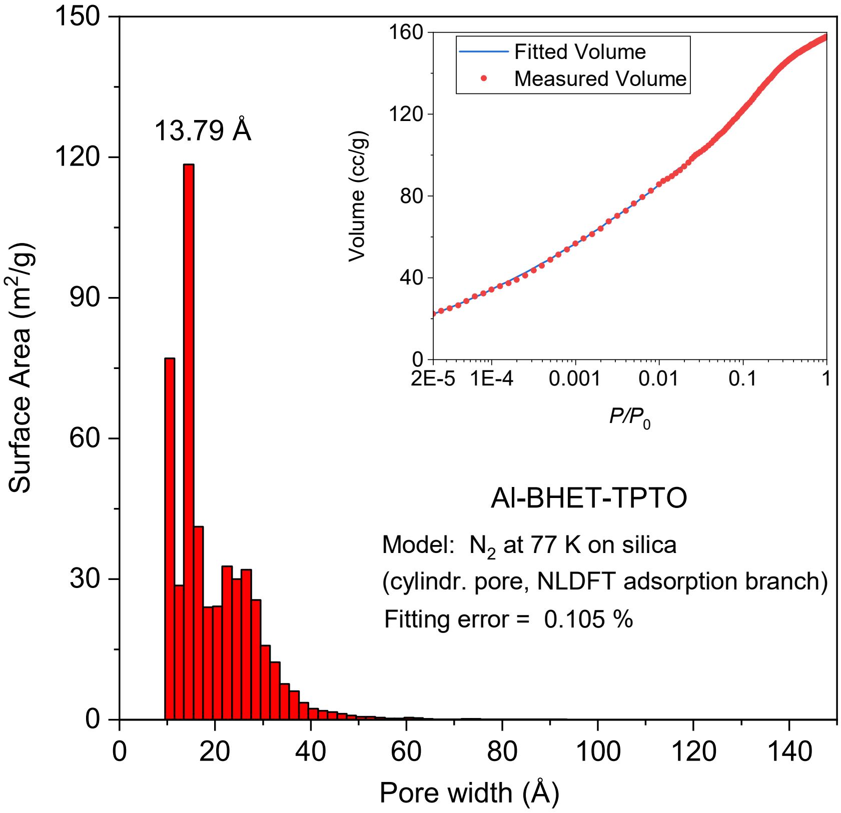

Supplementary Figure 29. Pore size distribution of Al-BHET-TPTO under 77 K nitrogen gas. Fitting of the N_2 adsorption isotherm of Al-BHET-TPTO showing its microporosity.

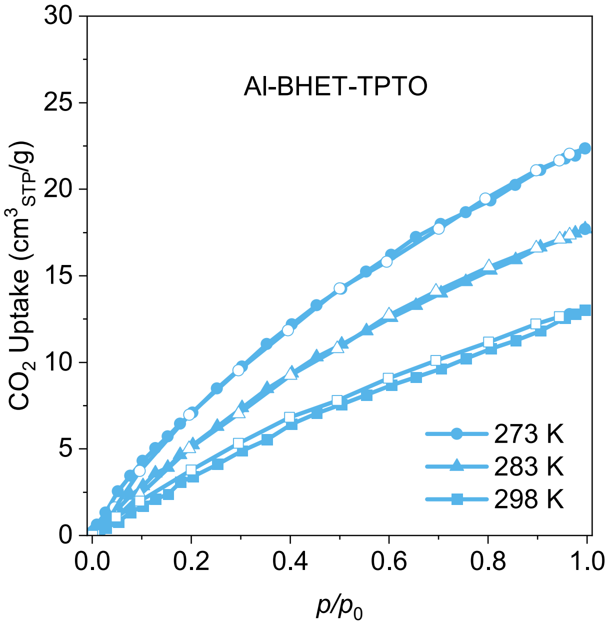

Supplementary Figure 30. CO_2 adsorption for Al-BHET-TPTO. The CO_2 uptake at different temperatures confirms the porosity of the Al-BHET-TPTO.

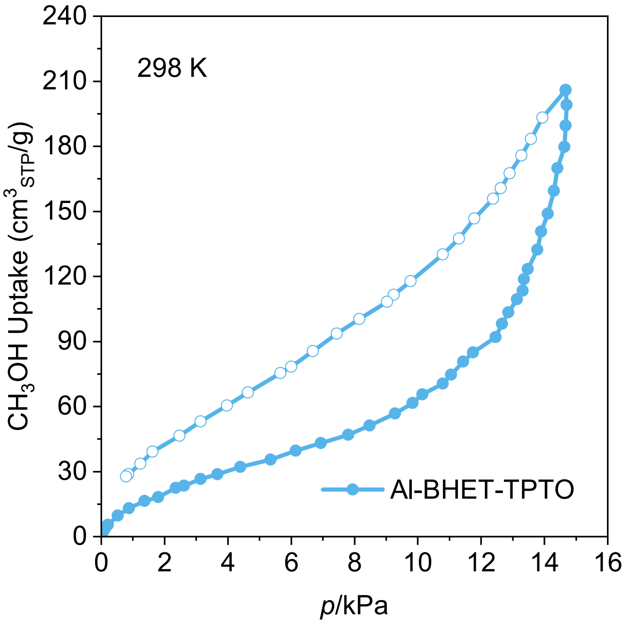

Supplementary Figure 31. 298 K methanol vapour adsorption of Al-BHET-TPTO. The methanol uptake showing the porosity of the Al-BHET-TPTO.

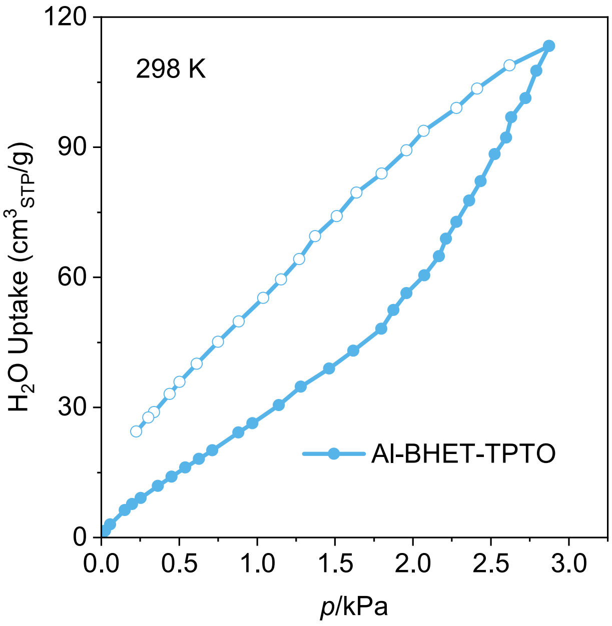

Supplementary Figure 32. 298 K water vapor adsorption of Al-BHET-TPTO. The water uptake showing the porosity of the Al-BHET-TPTO.

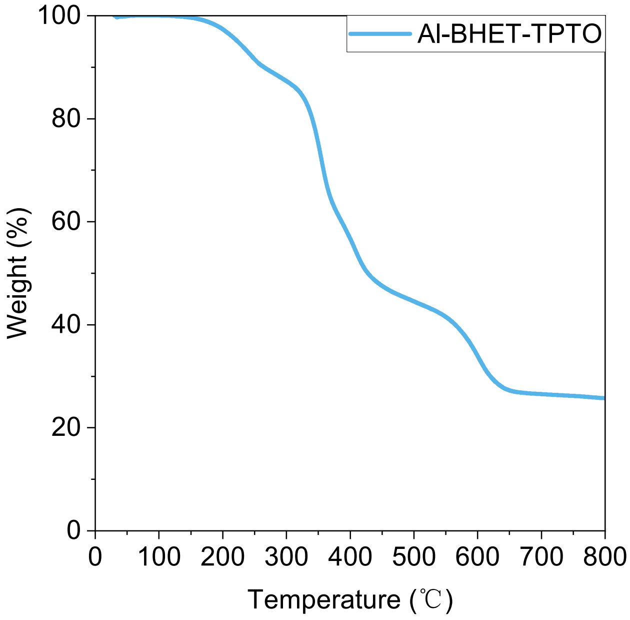

Supplementary Figure 33. TGA curve of Al-BHET-TPTO. The Al-BHET-TPTO shows thermal stability up to 150 °C without weight loss.

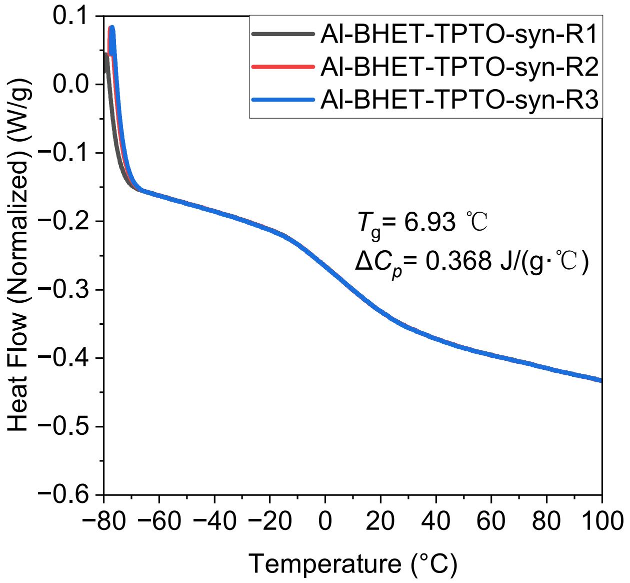

Supplementary Figure 34. DSC repeatability test for Al-BHET-TPTO-syn. The scan rate is 20 K/min.

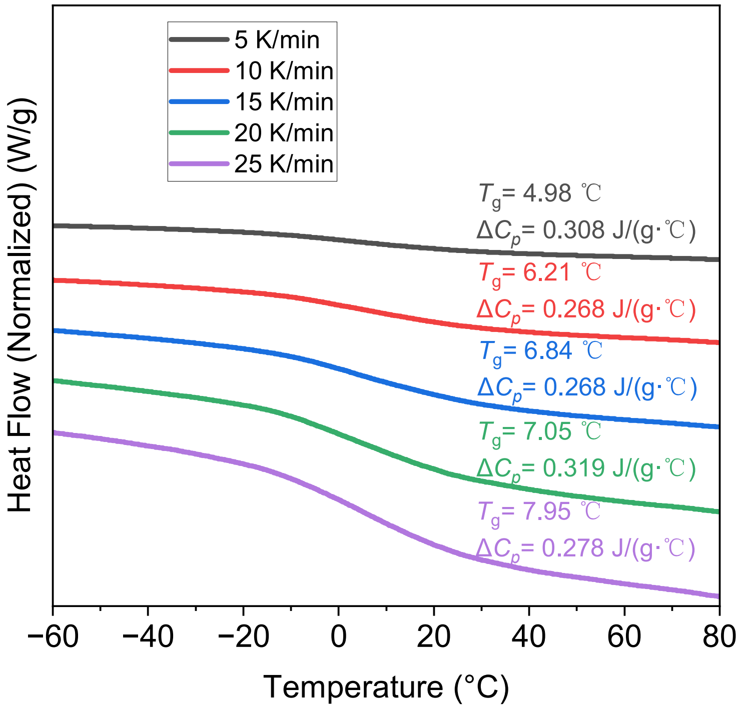

Supplementary Figure 35. DSC with different scan rate for Al-BHET-TPTO-syn. The increase of fictive temperature with higher scan rate confirms the presence of glass transition.

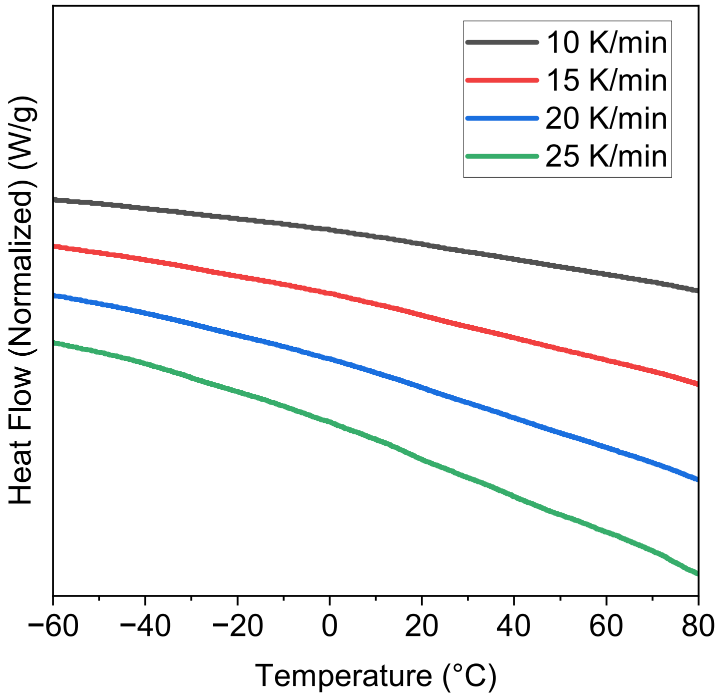

Supplementary Figure 36. DSC with different scan rate for Al-BHET-TPTO-ads. No glass transition is observed for scan rates varied from 10 K/min to 25 K/min.

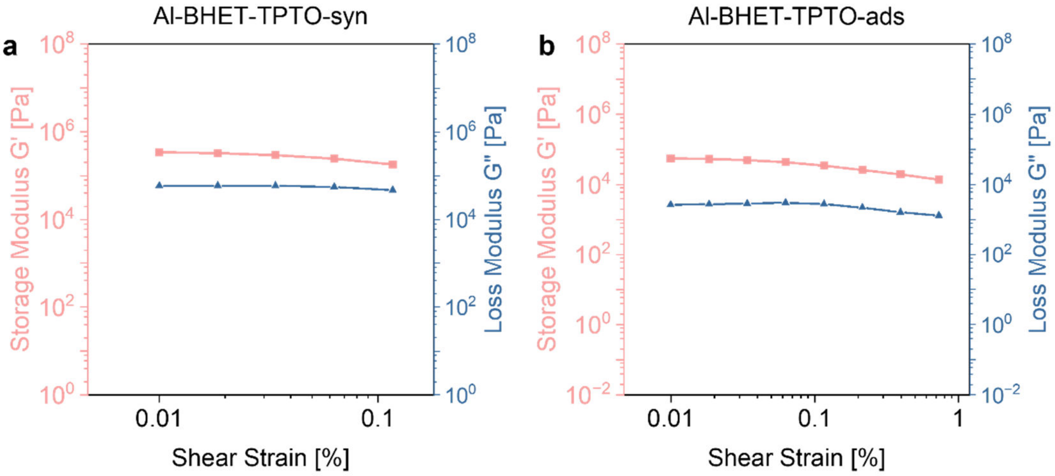

Supplementary Figure 37. Rheology measurement for Al-BHET-TPTO. a As-synthesized Al-BHET-TPTO. **b** Activated Al-BHET-TPTO. The suffix "syn" denote the as-synthesized sample, and "ads" denote the activated sample.

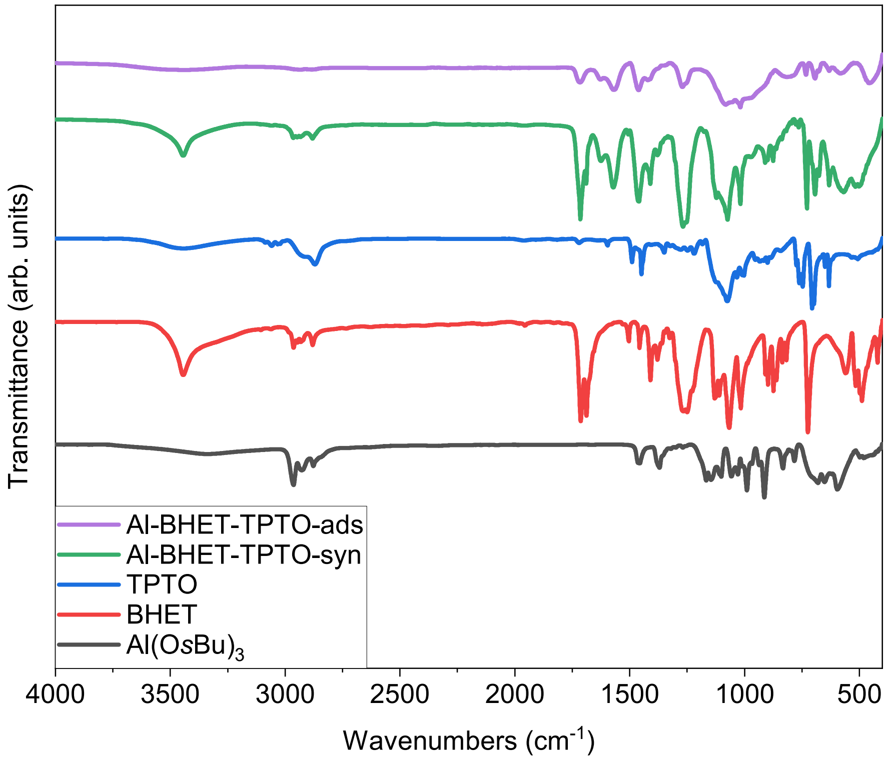

Supplementary Figure 38. Infrared spectrum of Al-BHET-TPTO. The diminished absorption above 3000 cm^{-1} shows deprotonation of the BHET linker.

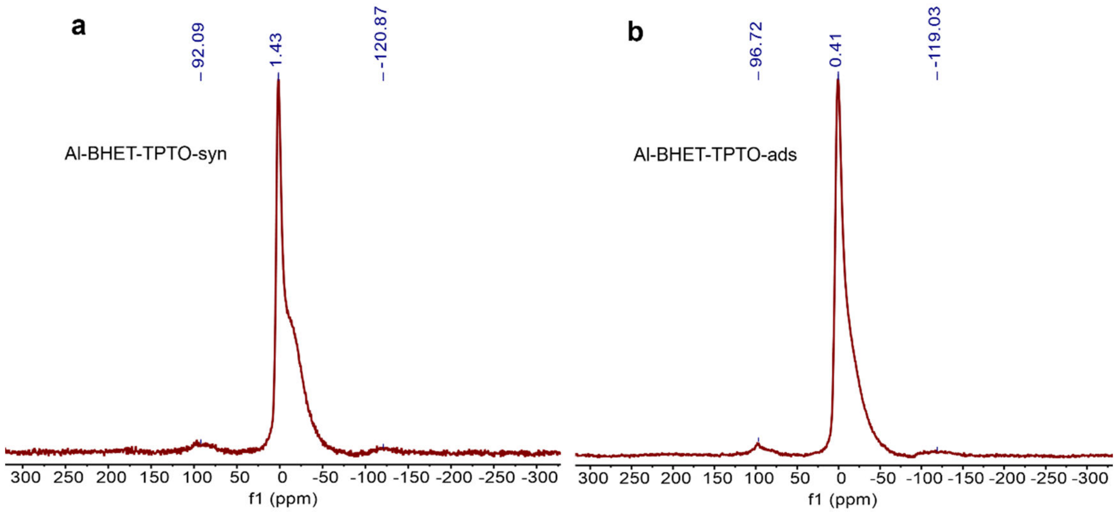

Supplementary Figure 39. ^{27}A1 CPMAS NMR spectra of Al-BHET-TPTO. a As-synthesized. **b** Activated. The suffix "syn" denote the as-synthesized sample, and "ads" denote the activated sample.

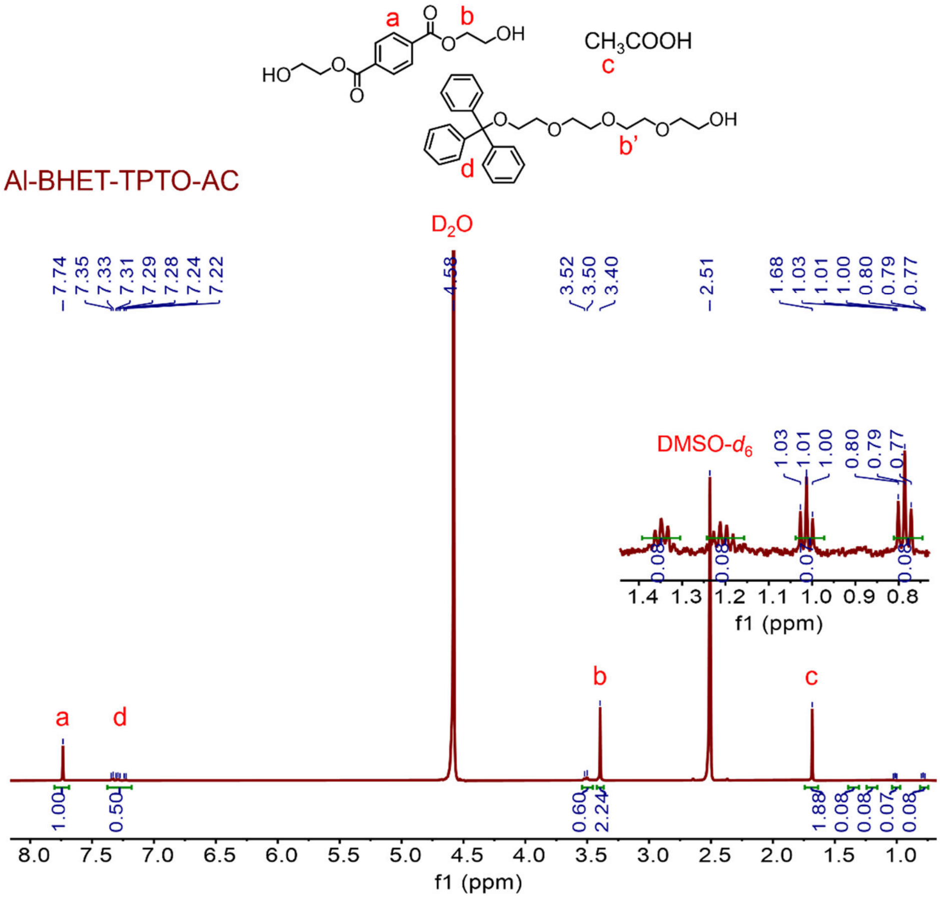

Supplementary Figure 40. Digested NMR spectrum of acetone-exchanged Al-BHET-TPTO-AC. The suffix "AC" denotes the sample after solvent exchange with acetone. ^{1}H NMR (500 MHz, DMSO-d_6) δ 7.74 (s, 4H), 7.41-7.16 (m, 15H), 3.51 (d, J = 10.7 Hz, 16H), 3.39 (s, 8H), 1.68 (s, 3H). molar ratio of BHET to TPTO is 7.5.

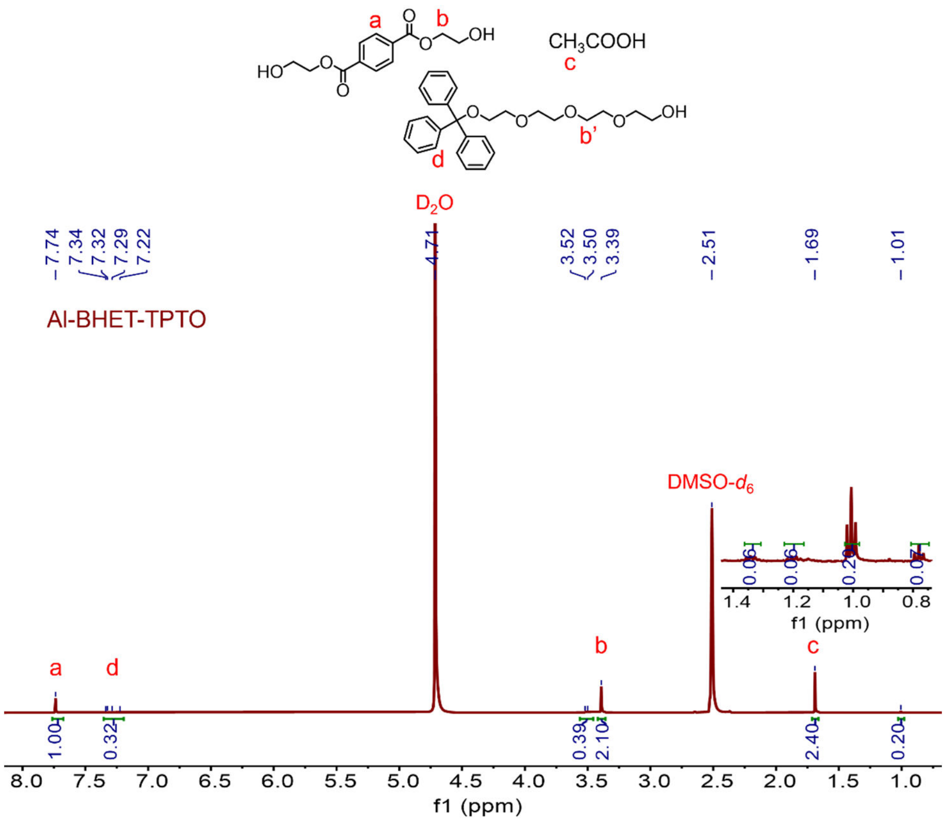

Supplementary Figure 41. Digested NMR spectrum of activated Al-BHET-TPTO. From the integrated peak area, the molar ratio of BHET: TPTO=11.72, substantially higher than the value initially in the solution.

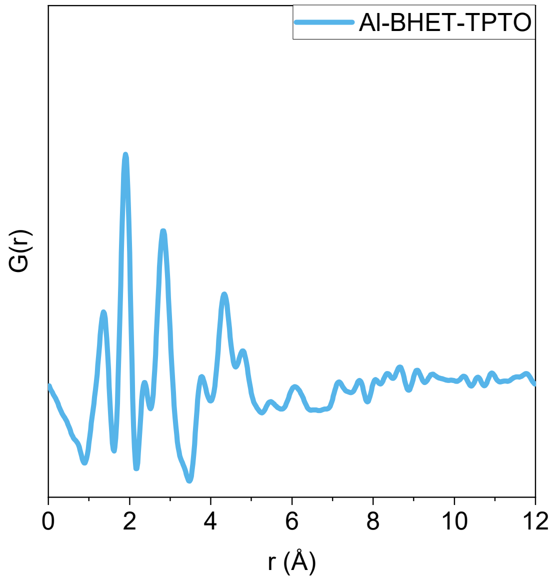

Supplementary Figure 42. Pair-distribution function of Al-BHET-TPTO. The short-range ordering is consistent with the structure of AlOCs.

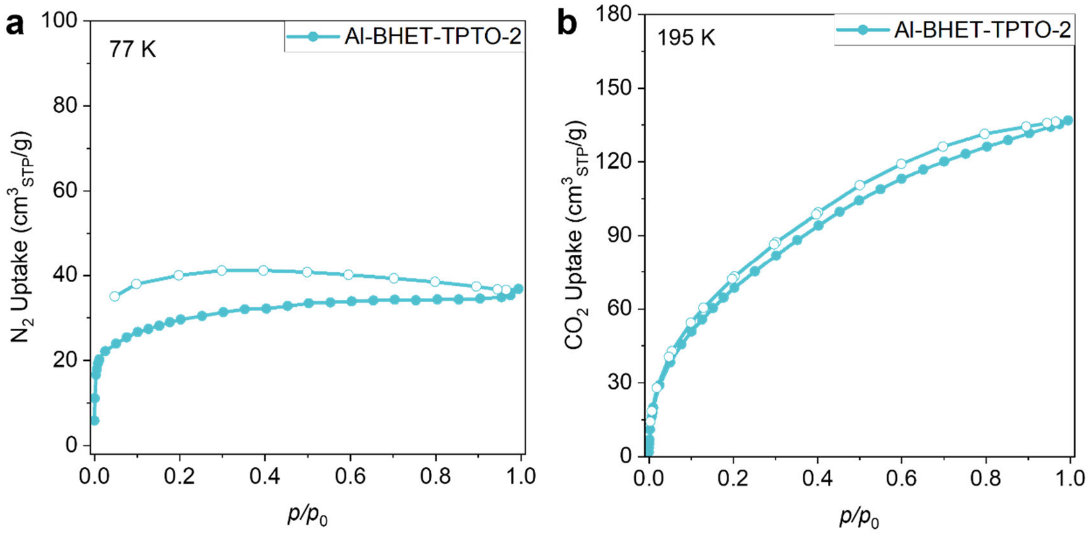

Supplementary Figure 43. Gas adsorption isotherms of Al-BHET-TPTO-2. **a** 77 K N_2 adsorption show lower uptake than Al-BHET-TPTO. **b** 195 K CO_2 showing similar uptake as Al-BHET-TPTO. The result shows that the Al-BHET-TPTO-2 has similar pore volume as Al-BHET-TPTO but smaller pore size.

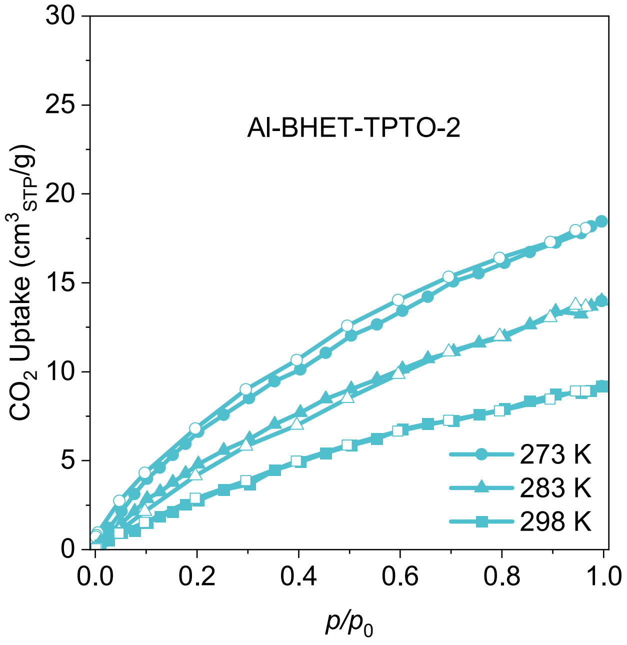

Supplementary Figure 44. CO_2 adsorption in Al-BHET-TPTO-2. The CO2 uptake at different temperatures confirms the porosity of the Al-BHET-TPTO-2.

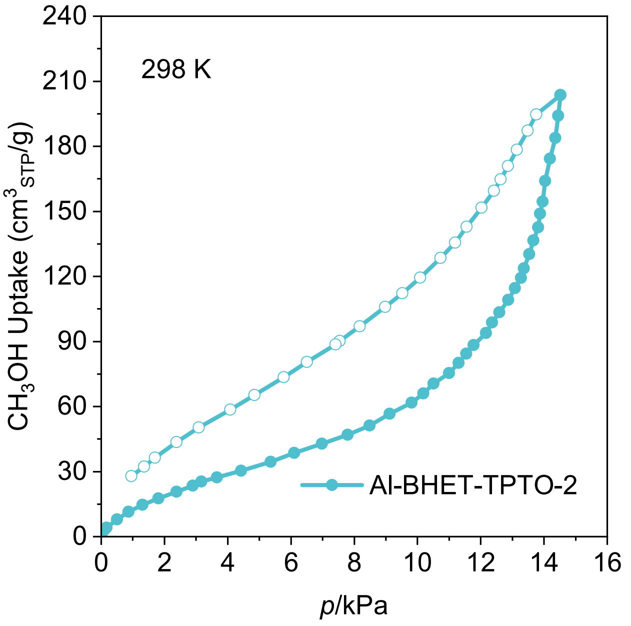

Supplementary Figure 45. 298 K methanol vapour adsorption isotherms of Al-BHET-TPTO-2. The methanol uptake showing the porosity of the Al-BHET-TPTO-2.

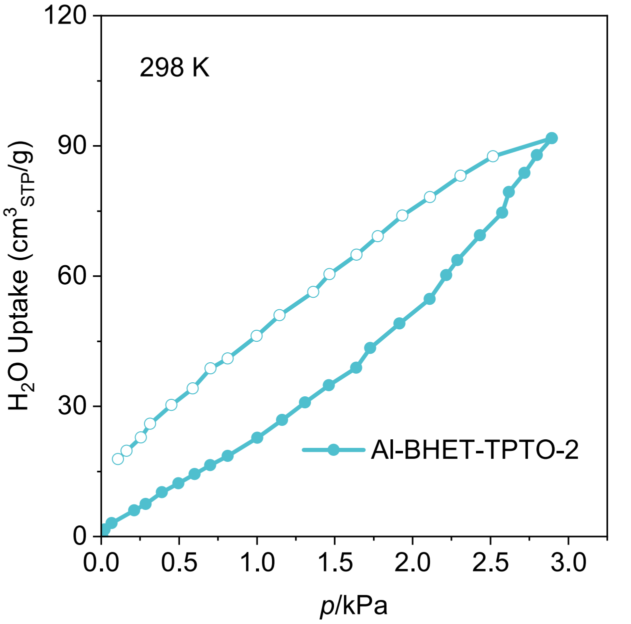

Supplementary Figure 46. 298 K water vapor adsorption of Al-BHET-TPTO-2. The water uptake showing the porosity of the Al-BHET-TPTO-2.

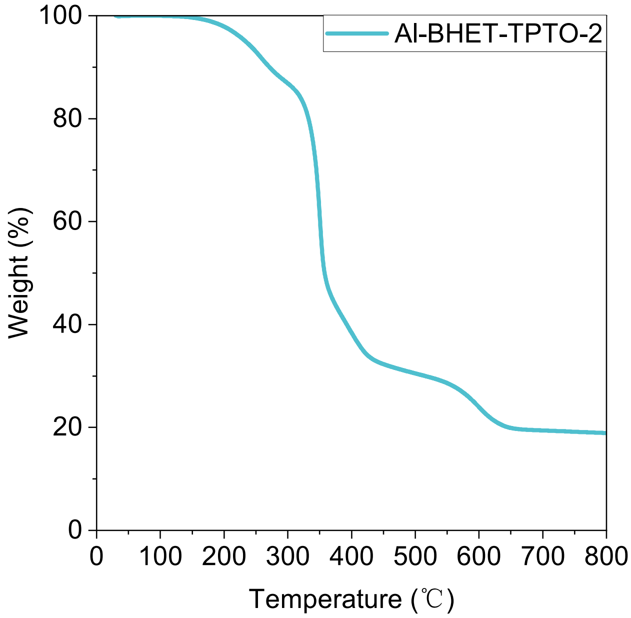

Supplementary Figure 47. TGA curve of Al-BHET-TPTO-2. The Al-BHET-TPTO-2 shows thermal stability up to 150 °C without weight loss.

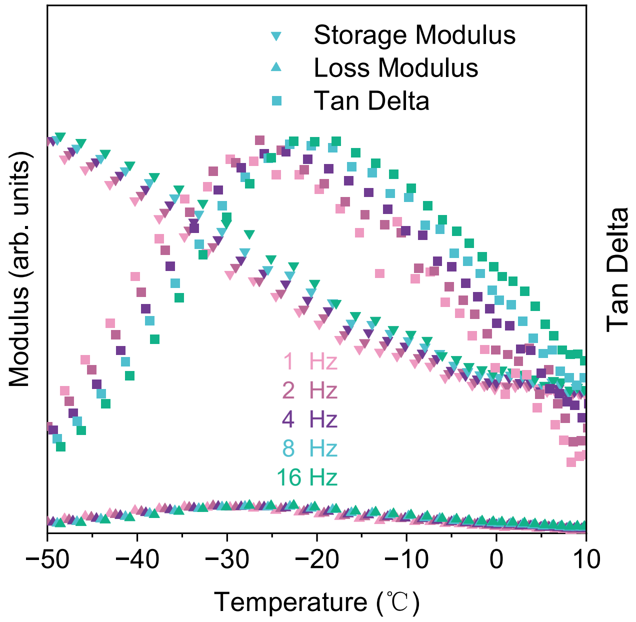

Supplementary Figure 48. Dynamic mechanical analysis graph of Al-BHET-TPTO-2-syn. The measurement frequencies are varied from 1 Hz to 16 Hz, which are denoted by different colors from pink to green. The storage modulus, loss modulus and tangent delta are denoted by inverted triangle, triangle and square, respectively.

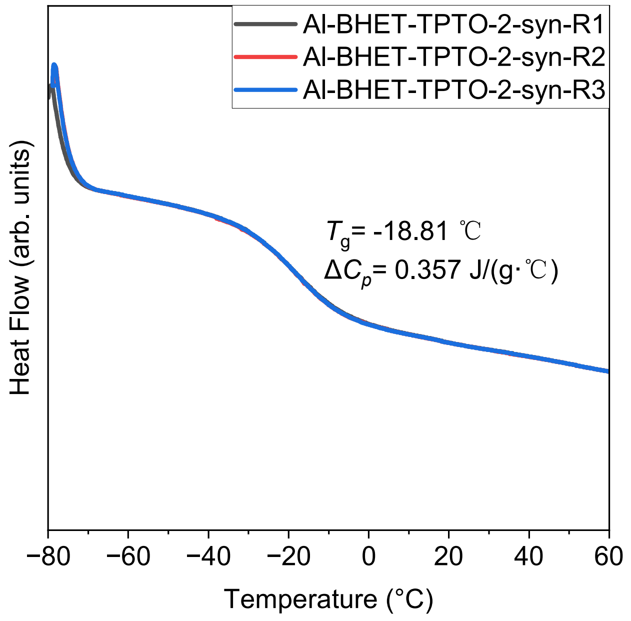

Supplementary Figure 49. DSC reversibility test for as-synthesized Al-BHET-TPTO-2-syn. The scan rate is 20 K/min. the suffix "syn" denote the as-synthesized sample, R1 to R3 denote the number of cycles.

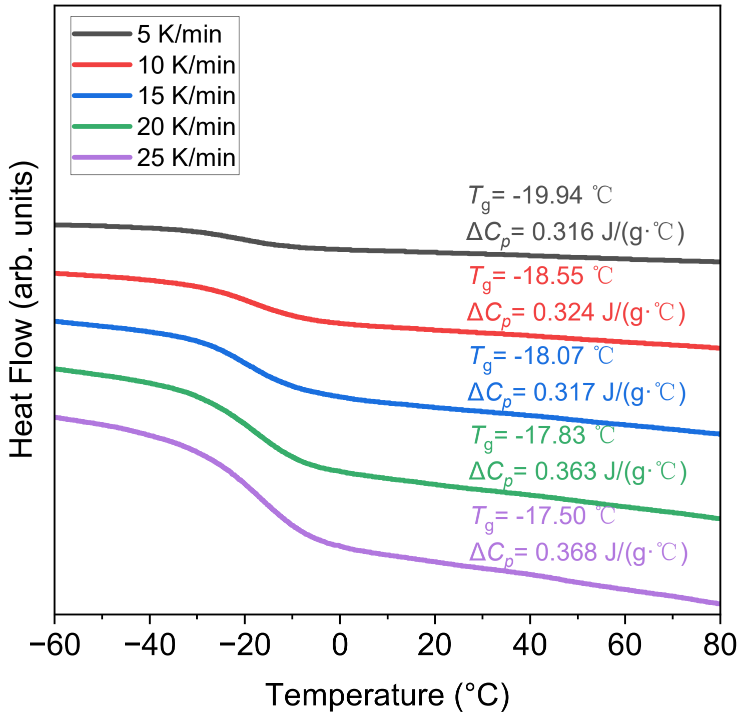

Supplementary Figure 50. DSC with different scan rate of Al-BHET-TPTO-2-syn. The increase of fictive temperature with higher scan rate confirms the presence of glass transition.

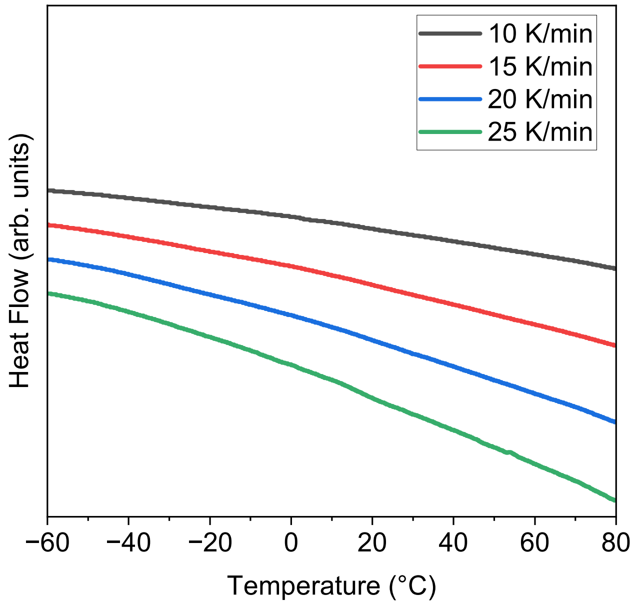

Supplementary Figure 51. DSC with different scan rate of Al-BHET-TPTO-2-ads. No glass transition is observed for scan rates varied from 10 K/min to 25 K/min.

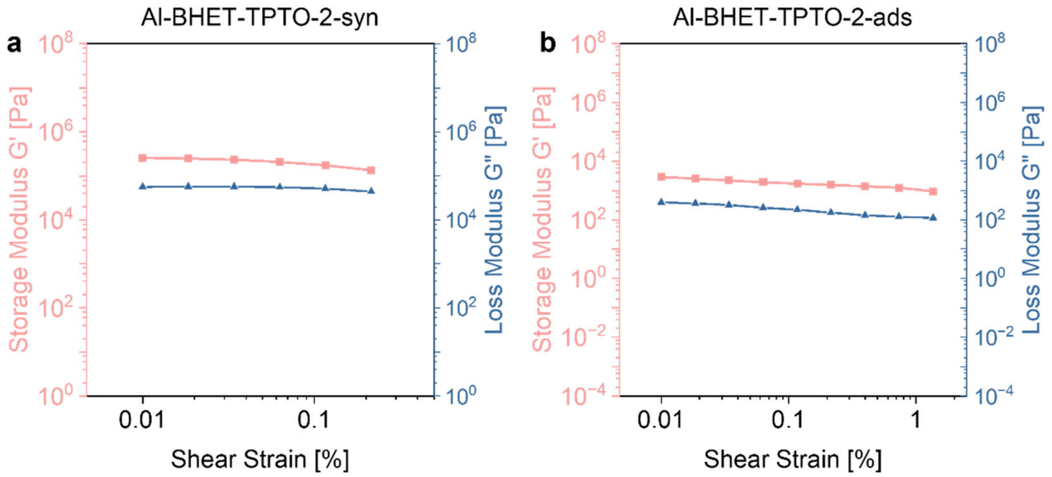

Supplementary Figure 52. Rheology measurement for Al-BHET-TPTO-2. a As-synthesized Al-BHET-TPTO-2. **b** Activated Al-BHET-TPTO-2. The suffix "syn" denote the as-synthesized sample, and "ads" denote the activated sample.

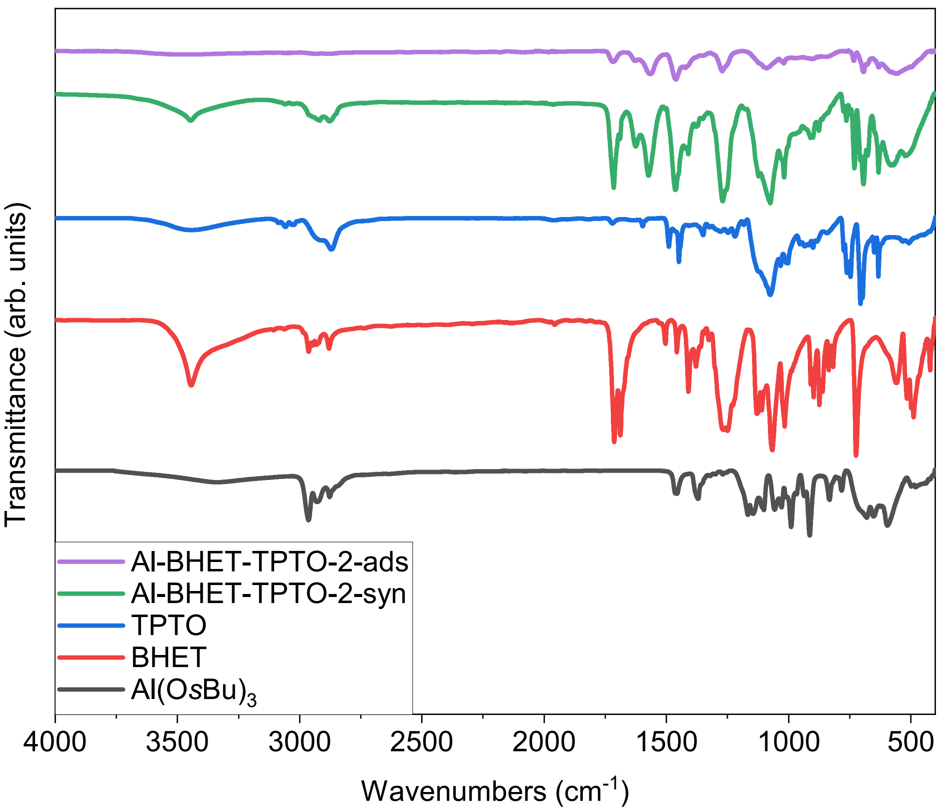

Supplementary Figure 53. Infrared spectrum of Al-BHET-TPTO-2. The diminished absorption above 3000 cm^{-1} shows deprotonation of the BHET linker.

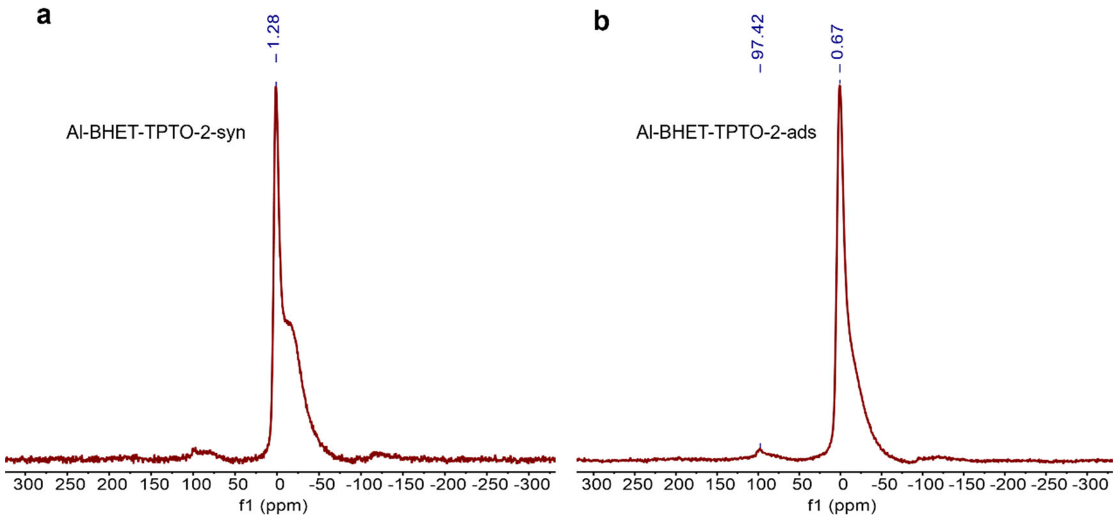

Supplementary Figure 54. ^{27}Al CPMAS NMR spectra of Al-BHET-TPTO-2. a As-synthesized. **b** Activated. Both spectra indicate the presence of Al in octahedral coordination environment.

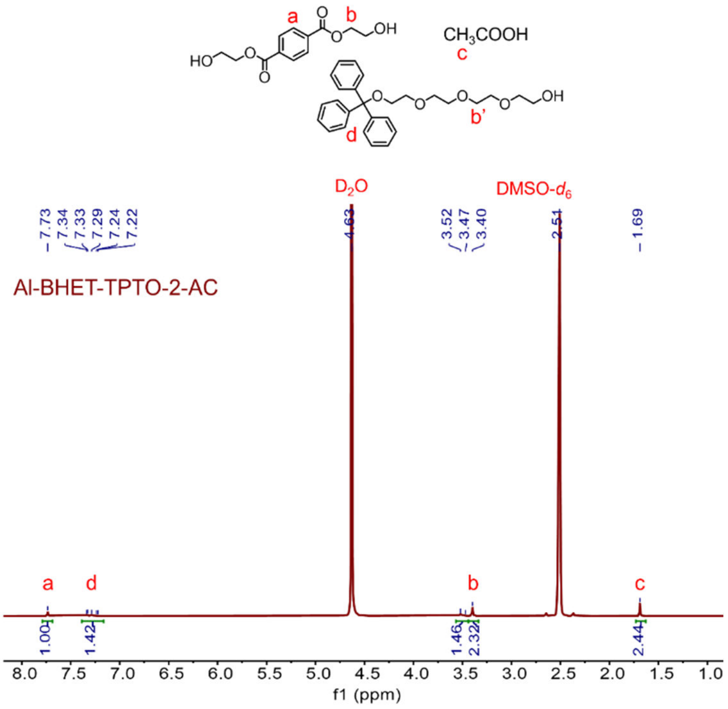

Supplementary Figure 55. Digestion nuclear magnetic resonance of Al-BHET-TPTO-2-AC. Peak integration give the molar ratio BHET: TPTO= 2.63.

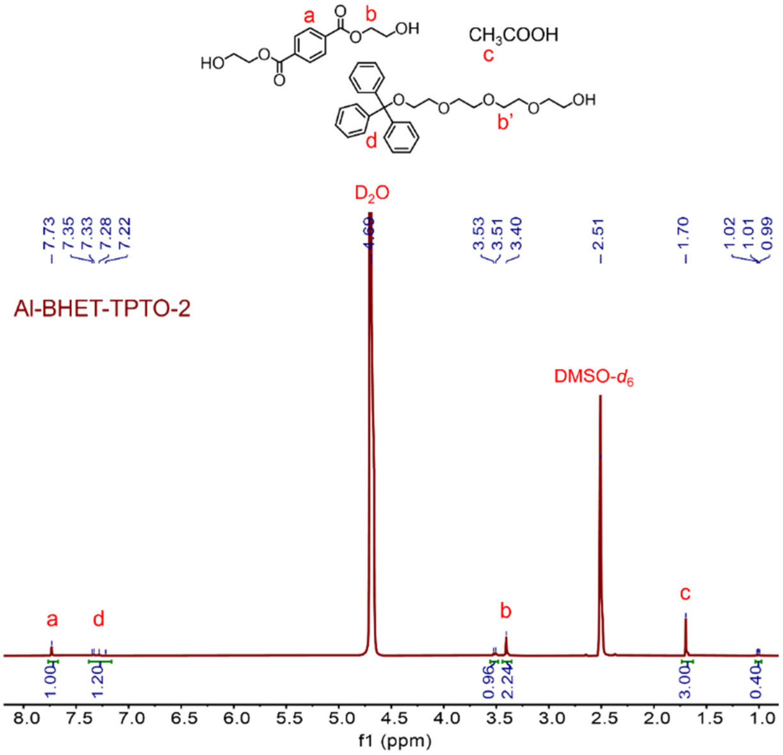

Supplementary Figure 56. Digestion nuclear magnetic resonance of activated Al-BHET-TPTO-2. Peak integration gives the molar ratio BHET: TPTO= 3.1.

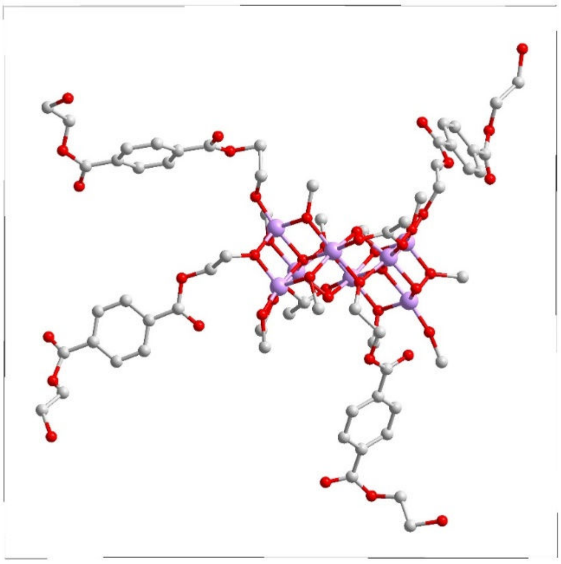

Supplementary Figure 57. Structure model of Al-BHET for pair-distribution function simulation. The simulated PDF is generated with this model using the I.S.A.A.C.S program (supplementary ref. 2). The structure model has its inorganic core adopted from AlOC-41, which is then attached to BHET as peripheral ligand. Periodic boundary condition was applied in the calculation for simplicity..

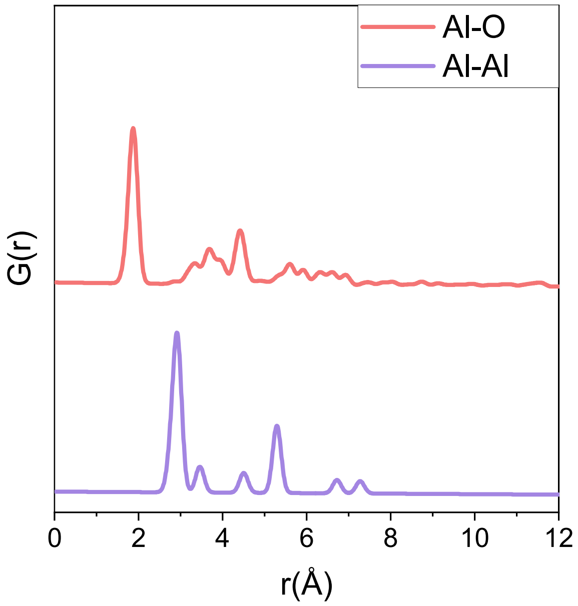

Supplementary Figure 58. Simulated G(r) for Al-Al and Al-O pair. The same model as supplementary fig. 57 is used for the simulation.

Supplementary References

1. Guang Lu, Chenlong Cui, Fengwei Huo, *et al*. Synthesis and Self-assembly of monodispersed metal-organic framework microcrystals. *Asian J*. **8**, 69-72 (2013).
2. Sébastien Le Rouxa and Valeri Petkova. ISAACS - interactive structure analysis of amorphous and crystalline systems. *J. Appl. Cryst.* **43**, 181-185 (2010).
